# Hypothermia After Cardiac Arrest as a Novel Approach to Increase Survival in Cardiopulmonary Cerebral Resuscitation: A Review

**DOI:** 10.5812/ircmj.17497

**Published:** 2014-07-05

**Authors:** Hassan Soleimanpour, Farzad Rahmani, Saeid Safari, Samad EJ Golzari

**Affiliations:** 1 Medical Education Research Center, Tabriz University of Medical Sciences, Tabriz, IR Iran; 2 Department of Emergency Medicine, Tabriz University of Medical Sciences, Tabriz, IR Iran; 3 Department of Anesthesiology and Critical Care, Iran University of Medical Sciences, Tehran, IR Iran; 4 Cardiovascular Research Center, Tabriz University of Medical Sciences, Tabriz, IR Iran

**Keywords:** Heart Arrest, Hypothermia, Out-of-Hospital Cardiac Arrest, Cardiopulmonary Resuscitation

## Abstract

**Context:**

The aim of this review study was to evaluate therapeutic mild hypothermia, its complications and various methods for induced mild hypothermia in patients following resuscitation after out-of-hospital cardiac arrest.

**Evidence Acquisition:**

Studies conducted on post-cardiac arrest cares, history of induced hypothermia, and therapeutic hypothermia for patients with cardiac arrest were included in this study. We used the valid databases (PubMed and Cochrane library) to collect relevant articles.

**Results:**

According to the studies reviewed, induction of mild hypothermia in patients after cardiopulmonary resuscitation would lead to increased survival and better neurological outcome; however, studies on the complications of hypothermia or different methods of inducing hypothermia were limited and needed to be studied further.

**Conclusions:**

This study provides strategic issues concerning the induction of mild hypothermia, its complications, and different ways of performing it on patients; using this method helps to increase patients’ neurological survival rate.

## 1. Context

According to American Heart Association (AHA) recommendations, cerebral resuscitation is the most important objective in advanced cardiopulmonary cerebral resuscitation (CPCR) (1). A long time has elapsed since replacement of the term cardiopulmonary resuscitation (CPR) with CPCR due to importance of preserving brain function in patients with cardiac arrest. One of the most important measures for the realization of cerebral resuscitation is the induction of mild hypothermia in patients with cardiac arrest (hypothermia after cardiac arrest or HACA) (2). In 2003, AHA recommended implementing HACA on all unconscious adult patients with return of spontaneous circulation (ROSC) after CPCR when initial rhythm was ventricular fibrillation (3); and announced that from 2010 onwards, the implementation of this important guideline on all the patients is mandatory (1).

Out-of-hospital cardiac arrest affects 250000 people annually in the United States. The overall survival of out-of-hospital cardiac arrest has been reported to be 6% (4). In the United Kingdom, 30000 people undergo CPR annually due to out-of-hospital cardiac arrest and only one of the twenty victims survives until hospital discharge (5). Based on the studies in Iran, a mortality rate of 90% has been reported for those undergoing CPR and only 7% of the survivors could be discharged from the hospital (6); however, the survivors of CPR are prone to severe neurologic complications (5). The short-term and long-term survival and improvement in prognosis of the patients after CPR are immensely related to rapid onset of CPR and related advanced cardiac interventions, which in fact, are the chains of the survival process; these processes include the diagnosis of the critical patients in order to prevent cardiac arrest, primary and rapid access to the patient, rapid onset of CPR and basic life support, timely defibrillation, advanced cardiac life support, and post-CPR care (6).

## 2. Evidence Acquisition

Articles used in this review were accessed from the available evidence on the HACA. The following key words were used to search databases: cardiac arrest; cardiopulmonary resuscitation (CPR); post CPR management; and hypothermia after cardiac arrest. Firstly, we searched for systematic reviews, evidence-based clinical practice guidelines, health technology assessments, and randomized controlled trials. In addition, in order to achieve a better conclusion, we used the following data bases and sites: Cochrane library and PubMed. 

In this manuscript we just reviewed the literature published from 1995 up to 2014. We included studies concerning mild induced HACA, CPR, post-CPR care, and studies performed in adults. Published studies in languages other than English or Farsi were excluded. To search the databases, we have used the keyword mentioned in the abstract of our article.

### 2.1. Analysis

**Figure 1.**
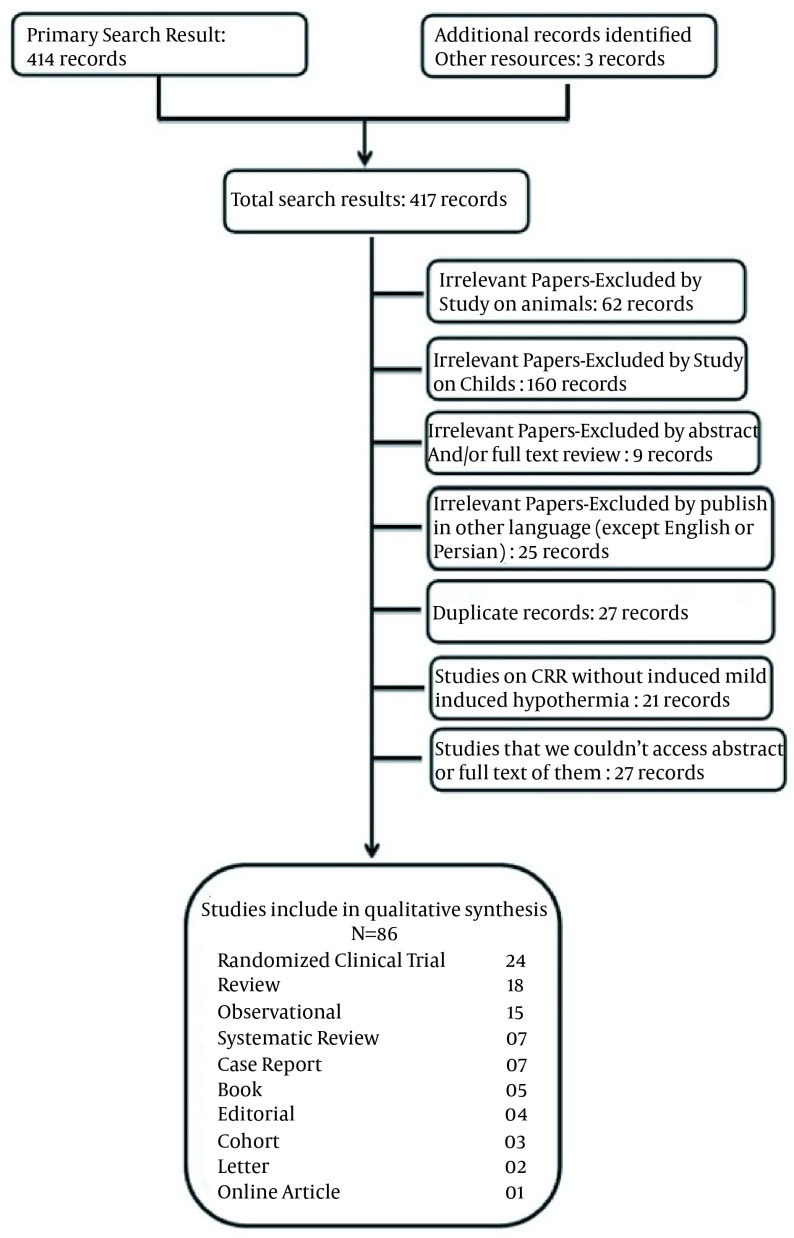
Flow Chart of Study

The search strategy resulted in 417 articles. The irrelevant papers based on the exclusion criteria (256) were excluded leaving 161 articles. A total of 79 articles were selected for further analysis including 21 randomized clinical trials, 17 review articles, 15 observational studies, four systematic reviews, seven case reports, five books, four editorials, three cohort studies, two letters, and one online article. A flow chart of our study is presented in Figure 1.

## 3. Results

### 3.1. Historical Perspectives

Undoubtedly, the subject of therapeutic hypothermia and post-resuscitation care after cardiac arrest cannot be imagined without its introducer, Professor Peter Safar (1924-2003, Vienna, Austria) who is known as the father of modern resuscitation. He was the world leading pioneer in the field of therapeutic hypothermia (2, 7).

The history of therapeutic hypothermia dates back to the 1950s. At that time, moderate hypothermia (32℃-28℃) was used in anesthesia for cardiac and brain surgery to protect the brain against ischemia. Later in 1960s, Dr Peter Safar proposed the use of moderate HACA within the algorithm of CPR. Side effects of moderate hypothermia were studied for a period of 25 years, the most important of which were shivering, vasospasm, increased plasma viscosity, increased hematocrit, hypocoagulation state, ventricular fibrillation, and reduced resistance to infection. Furthermore, induction of moderate hypothermia in patients and preserving the body temperature in this range during hypothermia was difficult. Thus, induction of moderate HACA was halted and research continued by inducing mild hypothermia on animal models. According to the results of previous studies, Safar et al. obtained good neurological outcomes from conducting hypothermia on animal models suffering from prolonged cardiac arrest (8-11). Then a number of clinical trials were conducted on mild HACA on humans, which had significant benefits in reducing mortality and improving neurological function.

In a study by Bernard et al. on 77 patients with out-of-hospital cardiac arrest in 2002, core body temperature of patients was reduced to 33℃ within two hours after ROSC and was kept at this temperature for 12 hours (Australian protocol) (12). The primary endpoint of this study was based on good cerebral function at discharge or referral to rehabilitation centers. In this study, 49% of patients in the HACA group and 26% in the control group had acceptable neurological outcomes. Mortality rate in the HACA and control groups was 51% and 68%, respectively, but the rate of complications in two groups was not significantly different (12).

In another study entitled "The Hypothermia After Cardiac Arrest (HACA)", conducted on 273 patients with out-of-hospital cardiac arrest and ten patients with in-hospital cardiac arrest (mostly surgical patients), core body temperature was reduced to 34℃ and 32℃ within four hours of ROSC and kept at this temperature for 24 hours (European/Austrian protocol). The primary endpoint of this study was to evaluate the proper cerebral function after cardiac arrest, i.e. normal cerebral function or adequate function to continue living independently and working part-time, and the secondary endpoint was to examine the incidence of complications within seven days or death within six months of cardiac arrest (13). In this study 55% of patients in the HACA group and 39% in the control group had acceptable neurological outcomes. Mortality rates in the hypothermia and control groups were 41% and 55%, respectively. Although hypothermic patients were more at risk in terms of the incidence of complications such as bleeding, pneumonia, and sepsis in comparison with controls, the differences were not statistically significant (13).

Further studies on the administration of HACA have been conducted in other conditions. HACA has been associated with desirable results in stroke patients, which could be due to decreased brain edema and intracranial pressure (ICP) (14-16). In addition, it has been implemented in pediatric patients; however, due to complications such as myocardial depression in induction phase and life-threatening complications in rewarming phase, some studies have not suggested it in pediatric cardiac arrest (17). In contrast, other studies have reported increased survival following HACA in children (18), which indicates the need for further studies in this field. Considering the benefits of HACA in out-of-hospital cardiac arrest, this approach is presently advised following myocardial infarction (MI); albeit, further confirming studies are required. In a 28-year-old patient, HACA was associated with favorable results (19, 20).

### 3.2. Pathophysiology of Neuronal Damage in Cardiac Arrest

Neuronal injury may occur during cardiac arrest, after ischemia, or after ROSC. During ischemia and hypoxia, loss of ATP production, Na^+^/K^+^ pump failure, cellular membrane breakdown, and activation of phospholipase lead to lipolysis, release of arachidonic acid, glutamate, and other toxic neurotransmitters, and increase in intracellular calcium (21, 22).

ROSC may cause delayed neuronal death as endothelial damage to arteries and cerebral parenchyma activates leukocytes and is followed by the release of cytokines and adhesion molecules. This will lead to the release of free radicals, proteases, tumor necrosis factor alpha, interleukins (IL-1, IL-6, IL-8, and IL-10), increase in vascular permeability, damage to blood brain barrier (BBB), and brain edema. Brain edema will also lead to increased oxygen diffusion inequality and decreased cerebral perfusion pressure, and consequently, ongoing hypoxia (22-25).

### 3.3. Mechanism of Hypothermia

For effective use of hypothermia in clinical settings, it is important to understand its mechanism and side effects. Formerly, it was thought that therapeutic effects of hypothermia were only through slowing down the metabolism and reducing the oxygen and glucose consumption in brain cells. Now it is known that the effect of hypothermia on improving neurological outcome is accomplished not only by the abovementioned procedures, but also through other various mechanisms (7, 8).

Several studies have shown that failure or success in HACA is dependent on the speed of induction, duration of cooling, rewarming rate of patient, and preventing complications (26-28). Since, different destructive mechanisms will result in various complications after cardiac arrest, required time to induce hypothermia and duration of hypothermia retention are different depending on the patient's condition (8).

It is important to consider the various protective mechanisms of hypothermia in achieving therapeutic objectives and improving neurological outcome; these mechanisms are discussed below:

1. When hypothermia was clinically used for the first time, it was hypothesized that its only protective effect was reducing the rate of brain metabolism (for every reduction in temperature degree [in ℃], brain metabolism reduces by 5% to 8%) leading to decrease in oxygen and glucose consumption by brain cells; however, it is not the only protective effect of hypothermia and other protective mechanisms of hypothermia are equally or more important (29-31).
2. Following ischemia/reperfusion (I/R) in resuscitated patients after cardiac arrest, cells may be necrotized and their function may change either completely or partially (7, 8). Several studies have shown that hypothermia can decrease apoptosis pathway and prevent damage leading to cell death (32, 33).
3. There is much evidence to suggest that hypothermia can prevent harmful and destructive processes in brain cells during ischemia or after reperfusion. Following ischemia, the level of high-energy metabolites (e.g. ATP and creatine phosphate) reduces in brain cells within seconds along with the interruption of the supply of oxygen to the brain, which results in the changes in cell metabolism from aerobic to anaerobic and increase in the intracellular concentration of inorganic phosphate, lactate, hydrogen, and calcium (23, 34, 35). Results from hypothermia induction on animal models have shown that if hypothermia is initiated at early stages of neuronal excitation caused by cardiac arrest, it can block the progress of neurological deterioration or even reverse it. With regard to the onset of hypothermia after ROSC, different times have been mentioned ranging from 30 minutes to six hours (8).
4. In different types of brain damage, a prolonged and specific inflammatory response is created within one hour of the I/R. Proinflammatory factors such as tumor necrosis factor alpha and IL-1 are secreted in copious amounts by astrocytes, microglia, and endothelial cells. The level of these factors increases after one hour of reperfusion and remains high for five days. This process leads to immune system stimulation. Active leukocyte cells pass through the BBB and lead to the accumulation of inflammatory cells in the affected brain. Simultaneously, the complement system is activated and causes activation of neutrophils and then monocytes and macrophages. The mentioned process often occurs in the reperfusion phase simultaneously with the production of free radicals (8, 36). Fortunately, the onset of cellular destructive stage, caused by immune system responses, is often delayed (more than one hour) and provides ample time for the induction of hypothermia (8, 37).
5. Another destructive process following I/R is the production of free radicals (i.e. superoxide, peroxynitrite, hydrogen peroxide, and hydroxyl radicals). These factors play an important role in determining whether the cell enters into the death process or restores its function. Inhibition of the free radicals production has a direct association with induced hypothermia; in other words, with decrease in temperature, the amount of free radicals decreases. Although hypothermia does not completely inhibit free radicals production, it can dramatically reduce their production and concentration and improve their performance (8, 38).
6. I/R damages the BBB and lead to brain edema. Treatment measures such as mannitol in stroke or traumatic brain injury may increase damages to the BBB. On the other hand, hypothermia can have a protective effect on BBB through reducing vascular permeability after the I/R phase by decreasing cerebral edema and significantly reducing the amount of damage. Furthermore, induction of hypothermia reduces the hemoglobin leakage from the vessels after traumatic brain injury (39, 40). In clinical practice, the amount of cerebral edema is determined by measuring the ICP. Clinical studies have shown that hypothermia may reduce ICP, increase life expectancy, and improve neurological outcome (8, 41).
7. I/R increases brain lactate levels. Levels of brain lactate are significantly reduced by hypothermia. In addition, glucose consumption in the brain is disrupted during I/R. Some studies in this area have concluded that hypothermia improves glucose consumption by the brain. Several studies have shown that induction of hypothermia during and after reperfusion increases the rate of metabolism improvement through storing high-energy phosphates (ATP) and reduces the accumulation of toxic metabolites (8, 22).
8. In healthy people, the brain temperature may be higher than core body temperature. This temperature difference increases after brain injury, ranging from 0.1℃ to 2℃. In a normal person, there is not a clear difference in temperature between different parts of the brain; however, this difference can increase after brain injury due to increased destructive activity in the affected areas. It is noteworthy that the increase in temperature of brain leads to an increase in ICP. Therefore, reducing brain temperature in patients with cardiac arrest following induction of hypothermia leads to decreased ICP and brain metabolism rate (8, 42, 43).
9. Different studies indicate that cardiac arrest and resuscitation are associated with increased clotting activity and lead to formation of fibrin and microvascular obstruction in the brain and heart. Administration of anticoagulant agents such as heparin or recombinant tissue plasminogen activator factor (r-tPA) improved blood flow and increased possible survival in animal studies. In addition, thrombolysis can increase the brain tolerance to ischemia. Initial studies on patients with cardiac arrest at the early stages of CPCR have shown that the use of thrombolytic agents may increase life expectancy and improve neurological outcome (7, 39). Since hypothermia affects the number of platelets, function of platelets and coagulation cascade, which causes slight increase in susceptibility to bleeding, the anticoagulant effect of hypothermia may have a protective effect on the central nervous and cardiac systems (8, 32, 44).
10. Some studies have shown that hypothermia can affect the secretion of factors from endothelium, e.g. endothelin, thromboxane A2 (TXA2), and prostaglandin E2 (PGE2), that affect cerebrovascular system and other vessels. Endothelin and TXA2 are vasoconstrictive while PGE2 is a vasodilator agent. TXA2 may also cause platelet aggregation. These factors play an important role in the regulation of local blood flow to the brain and the balance between them is essential to maintain homeostasis. Some studies have shown that this balance is disrupted due to ischemia-induced brain damage and a small increase in the production of TXA2 causes vasoconstriction, thrombosis, and hypoperfusion in the affected areas. Hypothermia can correct this imbalance (8, 45). Although some preliminary studies have shown that hypothermia can have desired effect on vascular factors and modification of cerebral blood flow, particularly in the damaged brain, the important effects of hypothermia should be investigated in more details. In addition to body temperature, local blood flow to the brain depends on many other factors including the presence or absence of autoregulation of cerebral blood flow, normal ventilation, and level of blood gases and other therapeutic agents such as mannitol and hypertonic saline (7, 8, 46).
11. Another important protective mechanism of hypothermia is increased tolerance to ischemia. Studies on animals have shown that hypothermia increases the tolerance of brain tissue to ischemia. Therefore, given the results of the effects of hypothermia, it is used in vascular surgeries, cardiovascular surgery, and neurosurgery (8, 47, 48).
12. No convulsive seizures occur frequently in individuals with different brain injuries. There are many evidences showing that the seizure activities cause significant increase in injuries to the damaged brain. Conducted studies have shown that hypothermia has a repressive effect on seizure activity and hence, has a protective effect on the central nervous system (7, 8, 49).

### 3.4. Complications of Hypothermia

 Hypothermia leads to various physiological changes in the body. The normal function of many enzymes is temperature dependent; therefore, many enzymatic reactions (e.g. drug metabolism), blood circulation, respiration, and coagulation system are impaired due to hypothermia (7, 37).

Although some changes made by hypothermia are physiological, they are not desirable in critically ill patients. Hence, it is necessary to prevent or treat these changes. On the other hand, some complications of hypothermia need no special treatment. For example, mild hypothermia causes bradycardia and decreased cardiac output, but requires no special treatment. In contrast, hypothermia leads to insulin resistance and decreased insulin secretion and therefore, increases the blood sugar level. This effect of hypothermia should be treated, because increased blood glucose levels has negative effects on the neurological outcome (7, 8, 49). In patients treated with HACA, hypothermia is usually performed in three steps: induction of hypothermia, maintenance (maintaining body temperature), and rewarming. Each of these steps is associated with specific problems that require treatment (8).

At the phase of induction of mild hypothermia, complications such as electrolyte abnormalities and impaired glucose metabolism may develop before reaching the target body temperature. These short-term complication usually make the patient’s clinical condition unstable and lead to complicated treatment of these disorders. At this stage, the risk of such complications can be reduced by rapid cooling of patients through a combination of different methods such as rapid surface cooling plus rapid infusion of cold intravenous fluids. When temperature reaches about 33.5℃, patient becomes stable and there will be little risk of loss of fluid or intracellular fluid shifts. At this time, the shivering of patient stops or significantly reduces and no substantial changes will occur in hemodynamic parameters. In addition, this stage requires frequent adjustments to the ventilator settings and changes in the dose of vasoactive medications (8, 50, 51).

At the maintenance phase, the risk of acute electrolyte abnormalities reduces; however, other risks such as pneumonia, wound infections, and bedsores should be considered at this stage. The stage of patient’s rewarming is associated with electrolytes shift from intracellular to extracellular compartments. This complication is reduced with slow and controlled warming; rapid warming of patient may lead to restart of destructive processes (3, 4, 8, 52-59).

### 3.5. Patients’ Inclusion and Exclusion Criteria for Induced Hypothermia After Cardiac Arrest

Inclusion and exclusion criteria for induced HACA are presented in Tables 1 and 2 (7, 8, 50, 60).

**Table 1.** Inclusion Criteria for Induced Hypothermia After Cardiac Arrest (3, 32, 44)

Inclusion Criteria ^a^
**Patients resuscitated with an initial rhythm of V-tach or VF and witnessed cardiac arrest**
**PEA (enough research is not conducted)**
**ROSC in less than 60 minutes**
**Patient does not follow commands (GCS ≤ 8) after ROSC**
**Age between 18 and 75 years**
**Interval of 5 to 15 minutes from collapse to the first attempt at resuscitation**
**Obtaining ethical consent**

^a^ Abbreviations: V-tach, ventricular tachycardia, VF, ventricular fibrillation; PEA, pulseless electrical activity; ROSC, return of spontaneous circulation; and GCS, Glasgow coma score.

**Table 2.** Exclusion Criteria for Induced Hypothermia After Cardiac Arrest (3, 32, 44)

Exclusion Criteria
**Absolute**
The order of "Do not resuscitate"
Pregnancy
Severe cardiogenic shock (systolic blood pressure < 90 mmHg or mean arterial pressure < 50 mmHg for longer than 30 minutes despite the use of vasopressor)
Core body temperature < 30°C (86°F)
Cerebral hemorrhage
Patients who have not been intubated.
**Relative**
Coma due to other causes (trauma, medical, stroke, etc.)
Life-threatening arrhythmias
ECG changes indicative of ischemia.
QT interval > 47 ms
Age < 17 years (consulting with pediatrician)
Elapse of more than six hours from cardiac arrest
Systemic infection/sepsis
Conditions predisposing to bleeding of unknown causes
The use of warfarin (its antidote should be considered)
Reduction of arterial oxygen saturation to less than 85%
Any condition that is an obstacle to treatment according to physician judgment
Coma before cardiac arrest

#### 3.5.1. Induced Mild Therapeutic Hypothermia After Cardiac Arrest

After successful resuscitation of patients with cardiac arrest, in the first 15 minutes before implementing hypothermia protocol, the following measures should be taken:

Cardiopulmonary condition of patient must be stable; mean arterial blood pressure should be kept over 75 mmHg and if necessary, a vasopressor should be administer; the arterial oxygen saturation should be over 98%; initial neurological examination should be taken from the patient. These examinations include assessing pupils, corneal reflex, oculocephalic reflex, motor response following severe stimulation, and determining Glasgow Coma Score; vital signs and body temperature should be controlled; and sedatives and muscle-relaxant agents for the patient’s ease and relief should be administered (including midazolam, propofol, fentanyl, vecuronium, atracurium, rocuronium, and pancuronium) (36, 50, 61-72). In addition, in a study by Nielsen et al. target temperatures of 33℃ and 36℃ were compared on unconscious survivors following cardiac arrest of cardiac origin and they concluded that neurological outcome had no significant difference following these settings (62).

### 3.6. Protocol Selection

**Figure 2.**
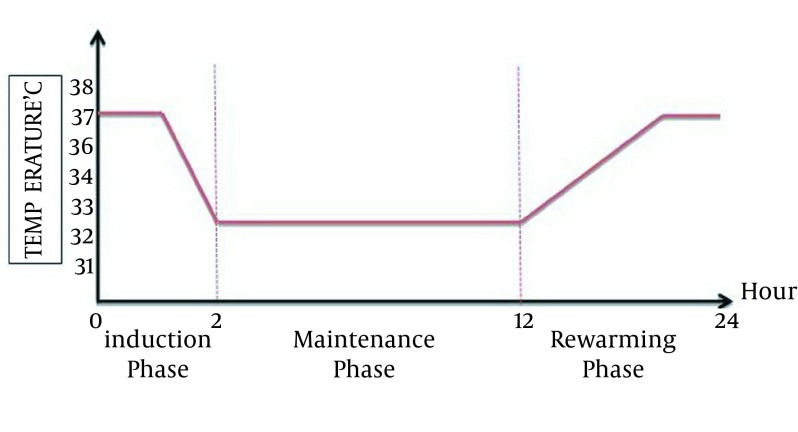
Australian Protocol; Desired Body Temperature at Three Phase of Mild Induced Hypothermia After Cardiac Arrest (12)

**Figure 3.**
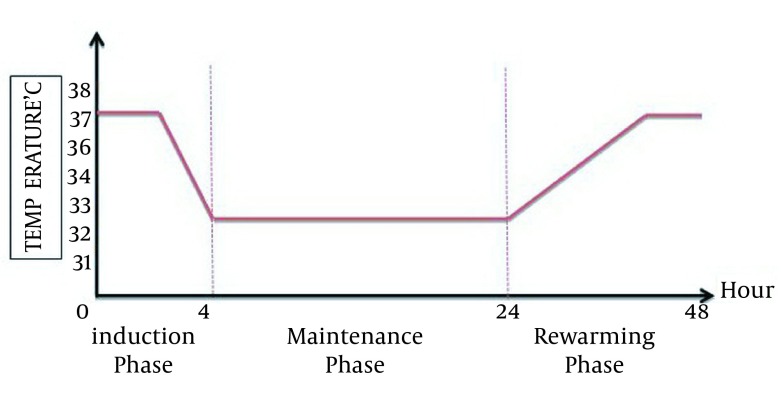
European/Austrian Protocol; Desired Body Temperature at Three Phase of Mild Induced Hypothermia After Cardiac Arrest (13)

1. Australian protocol: Core body temperature of patients should be reduced to 33℃ within two hours of ROSC and be kept at this temperature for 12 hours (Figure 2) (12, 13).
2. European/Austrian protocol: Core body temperature of patients should be reduced to 32℃ or 34℃ within four hours after ROSC and be kept at this temperature for 24 hours (Figure 3).

There are several techniques to lower the temperature that can be used depending on the availability or the experience of physician (8).

### 3.7. Types of Cooling Methods

#### 3.7.1. Cooling of the Body Surface

The simplest technique for HACA is to use ice packs on the head, neck and patient's body. There is another device called EMcools pad (Emergency Medical Cooling System) which includes a series of pads made of a combination of graphite and water. The inner layer of these pads is composed of a hydrogel (consistent with the patient's skin) and binds directly to the patient's skin resulting in the transfer of heat to the body. Rate of heat loss in this way is 2.9℃/h (8, 13, 73).

#### 3.7.2. Large Volume of Intravenous Cold fluids

A simple and inexpensive technique for induced mild HACA is the rapid infusion of a high volume of cold intravenous normal saline and Ringer's lactate fluids (40 mL/kg of fluid at 4℃) (5, 47). This method is a simple technique for cooling patients during CPR at the scene in prehospital setting. Although it has not yet been studied on humans, the studies on animals have indicated its effectiveness (12, 73).

#### 3.7.3. Intravascular Cooling

Different types of intravascular closed-end catheter are currently available for induction and retention of HACA. These catheters are usually placed in the venous system (femoral vein or inferior vena cava) and they contain a liquid circulating system with controlled temperature. Cooled liquid is transferred to the catheter through a heat exchanger system using a pump and flows inside it and thus, the core body temperature is reduced to the target temperature (62, 73, 74).

#### 3.7.4. Extracorporeal Membrane Oxygenation or Cardiopulmonary Bypass

Extracorporeal membrane oxygenation (ECMO) or cardiopulmonary bypass (CPB) system consists of a large intravascular catheter (usually venous catheter), blood pump, and heat exchange system that leads to a rapid and precise control of core body temperature; however, it is very expensive and needs an experienced and trained specialist. In this method, the patient should fully receive anticoagulation before connection to the device. Therefore, it is not used commonly in emergency and critical care units (7).

#### 3.7.5. Transnasal Cooling System (Rhino Chill)

The equipment of this device consists of a 12 kg backpack and contains a disposable nasal catheter, a control unit, and a tank containing 2 L of a coolant and an oxygen compartment. Mixture of oxygen and coolant is delivered to the patient through a tube connector and a 10 cm nasal catheter is placed along the base of the nasal cavity through nostrils and the coolant is sprayed into the nasal cavity from the distal end of the catheter (7).

#### 3.7.6. Pharmacological Techniques

A neurotensin analog was recently discovered. Following intravenous injection, the rapid induction of hypothermia in a matter of minutes without the need for sedation or anesthesia. In addition, with the elimination of neurotensin from the body (after 24 hours), the core temperature rises without the need for external heat; however, the safety of these drugs in humans is not known yet (8, 75).

#### 3.7.7. Ice-Cold Perfluorocarbon Ventilation

 Slowly injecting a high volume of cold perfluorocarbon into the patient’s lung will lead to a rapid drop in temperature and also proper oxygenation and ventilation. So far, this method has been studied only in animals (7, 8).

#### 3.7.8. Lavage of Body Cavities

Lavage includes gastric lavage (500 mL of the cool liquid every ten minutes), bladder lavage (300 mL of cold Ringer’s serum every ten minutes) and peritoneal lavage (2 L of 10℃ Ringer's serum is slowly poured into the peritoneal cavity and is then drained after five minutes) (76, 77).

#### 3.7.9. Brain Cooling

To avoid the adverse effects of hypothermia on body, selective brain cooling is preferred. Although no effect of hypothermia on body is found in this method, the side effect of carotid artery puncture in an emergency situation makes this method almost impractical (77).

#### 3.7.10. Cooling the Heart for the Intervention

Current opinion is that the rapid establishment of reperfusion together with the ST elevation MI leads to reduced size of the infarct and infract-related complications; however, reperfusion itself results in irreversible myocardial injury, which is called reperfusion injury. Empirical studies have shown the benefits of induction of hypothermia before reperfusion in MI, in reduction of the size of infarct area and making microvascular injuries, are limited. If hypothermia is initiated in the next phase of establishing reperfusion, it will have no effect on reducing the size of the infarct area. Two clinical trial studies have shown that the induction of hypothermia using intravascular catheters has not been effective in reducing the size of the infarct area. The reason is that the relevant target temperature has not been obtained before reperfusion. Another Study rapidly cooled the patients with MI before establishing the reperfusion using the method of cold saline administration and intravascular cooling. After establishing reperfusion, the size of myocardium at risk in hypothermic patients was reduced by 38% in comparison with the control group (78, 79).

### 3.8. Protocol of Rewarming the Patients

Depending on the Australian or European/Austrian protocol used for hypothermia, rewarming the patients should start after 12 or 24 hours after the onset of cooling, respectively. Rewarming is performed in passive or active ways. In passive rewarming, only coolant factor (ice packs, cold fluid, etc) is removed and patient is left to be automatically warmed. The rate of temperature rise during the warming phase should not exceed 0.25℃ (0.5℉) per hour (7, 8).

## 4. Conclusions

The induction of mild HACA is a new and novel approach which improves the neurological outcomes in unconscious adult patients with cardiac arrest; however, studies on the complications of hypothermia or different methods of induction are limited and further studies are required. This procedure has been approved by the AHA and it is mandatory for illegible patients with cardiac arrest.
